# Classification of Boston Doctors

**Published:** 1869-06

**Authors:** 


Classification of Boston Doctors.—The editor of the Boston *Med. & Surg. Journal* has been taking the census of the profession in his village, and finds 574 claimants of the title. Of these, 275 belong to the regular army, 13 are eclectics, 46 are skirmishers who load with infinitesimal globules, 60 are attached to the crinoline department, and 180 are bushwhackers. Among the latter are electric, botanic, magnetic, sympathetic, mesmeric, clairvoyant and cancer doctors, 2 "natural" bone-setters, 1 who practices "natureopathy," and 1 "Baunchedismus."

